# Genomic Characterization of Antimicrobial Resistance and Evolution Mechanism of Bacteria

**DOI:** 10.3390/antibiotics14090945

**Published:** 2025-09-18

**Authors:** Daniel Gyamfi Amoako, Linda Antionette Bester

**Affiliations:** 1 College of Health Sciences, University of KwaZulu-Natal, Durban 4000, South Africa; 2 Department of Pathobiology, University of Guelph, Guelph, ON N1G 2W1, Canada; 3 Department of Biochemistry and Microbiology, Faculty of Science, Engineering and Agriculture, University of Venda, Thohoyandou 0950, South Africa

Antimicrobial resistance (AMR) continues to rank among the most pressing global health threats, frequently referred to as a “*silent pandemic*” that undermines decades of progress in infectious disease control while jeopardizing both human and animal health [1]. The relentless emergence of resistant pathogens is fueled by the overuse and misuse of antimicrobials in clinical, veterinary, and agricultural settings, coupled with the remarkable genetic adaptability of bacteria via mechanisms like horizontal gene transfer. In this context, the genomic era has opened unprecedented avenues for discovery. Whole-genome sequencing and metagenomics now enable researchers to move beyond phenotypic observations to dissect the molecular blueprints of resistance determinants. These technologies illuminate how resistance determinants, virulence factors, and other mobile genetic elements (MGEs) interact to shape pathogen fitness and epidemiology across human, animal, and environmental reservoirs [2]. Furthermore, the integration of real-time genomic surveillance combining diagnostic microbiology, pathogen sequence data, and One Health collaboration offers timely insights for clinical practice and public health policies aimed at mitigating AMR spread [3].

This Special Issue assembles eleven diverse contributions from 97 authors, collectively enriching our comprehension of the molecular, evolutionary, and ecological dimensions of AMR. The studies included span various bacterial taxa, clinical and agricultural contexts, and geographic regions, highlighting both universal trends and local particularities in resistance evolution.

Several studies emphasize the critical role of One Health strategies in monitoring resistance across the farm-to-fork continuum and primary healthcare settings. Abdalla et al. employed genomic surveillance in South African intensive pig farming systems, revealing a broad spectrum of plasmid-borne resistance and virulence genes disseminated throughout the production chain from farms to transport and abattoirs, underscoring potential public health risks. Complementarily, Wolde et al. characterized extended-spectrum *β*-lactamase (ESBL)-producing *Escherichia coli* isolates from Ethiopian primary healthcare patients, uncovering notable genetic diversity, high-risk sequence types, and transferable plasmids, highlighting the mobility of resistomes within community health settings.

Comprehensive genomic and phenotypic analyses reveal the coexistence of AMR genes and virulence factors across multiple bacterial pathogens. Fahmy et al. investigated *Salmonella enterica* isolates from cattle, identifying multidrug resistance, robust biofilm formation, and diverse virulence determinants that enhance persistence and zoonotic potential. Hetsa et al. studied bloodstream infections caused by *Staphylococcus aureus* in South Africa, encompassing methicillin-resistant and susceptible strains, and reported complex resistance profiles coupled with variable virulence gene content, raising significant treatment challenges. Likewise, Yaikhan et al. examined clinical *Enterobacteriaceae* isolates from Thailand, unveiling multidrug resistance alongside novel bacterial species and diverse plasmid-mediated resistance mechanisms. In parallel, Er et al. analyzed bacteriocin-producing *E. coli* from healthy individuals, discovering strains co-harboring virulence and resistance elements, cautioning that such gut colonizers could serve as cryptic reservoirs of AMR.

The evolutionary plasticity of bacterial resistance is vividly illustrated in studies dissecting MGEs and genomic adaptations. Ishihara et al. traced the dissemination of *pESI-like* megaplasmids in *Salmonella Schwarzengrund*, demonstrating how structural variations in these large plasmids drive the spread of multidrug resistance among foodborne pathogens. In Hungary, Tóth et al. examined *E. coli* ST131 bloodstream isolates, revealing dynamic chromosomal integration of *β*-lactamase genes and plasmid rearrangements underpinning the global success of this high-risk clone. Watanabe et al. provided mechanistic insights into the regulation of *Sed-1 β*-lactamase in *Citrobacter sedlakii*, showing that single-point mutations and regulatory disruptions can transform otherwise susceptible strains into extended-spectrum *β*-lactamase producers.

The clinical ramifications of persistent AMR and therapeutic challenges are reflected in research addressing chronic infections and novel antimicrobials. Belkova et al. investigated *Pseudomonas aeruginosa* isolates persisting in cystic fibrosis patients under long-term therapy, revealing complex resistance trajectories, including instances of partial re-sensitization, highlighting the dynamic nature of resistance adaptation in chronic infections. Jayaweera et al. evaluated the novel fluoroquinolone WQ-3810 against resistant *Mycobacterium avium* strains, demonstrating significantly enhanced efficacy compared to existing agents and promising synergy in combination therapy.

The contributions in this Special Issue reveal AMR as both a scientific challenge and a global systems crisis. Evidence from agricultural, clinical, and community settings shows how the mobility of resistance determinants, often linked to virulence, reshapes bacterial populations and undermines health. Genomics is indispensable for uncovering hidden pathways of dissemination and persistence. Beyond documenting resistance, these studies underscore two priorities: advancing novel therapeutics and embedding genomic surveillance within One Health frameworks. Achieving impact requires global commitment to data sharing, harmonized surveillance, and policies that translate genomic insights into coordinated interventions aimed at curbing the trajectory of the AMR crisis.
